# How cytoskeletal proteins regulate mitochondrial energetics in cell physiology and diseases

**DOI:** 10.1098/rstb.2021.0324

**Published:** 2022-11-21

**Authors:** Tanya Solomon, Megha Rajendran, Tatiana Rostovtseva, Livia Hool

**Affiliations:** ^1^ School of Human Sciences, The University of Western Australia, Crawley, Western Australia, Australia; ^2^ *Eunice Kennedy Shriver* National Institute of Child Health and Human Development, National Institutes of Health, Bethesda, MD, USA; ^3^ Victor Chang Cardiac Research Institute, Darlinghurst, Sydney, New South Wales, Australia

**Keywords:** mitochondria, voltage-dependent anion channel, cytoskeleton, network, metabolic activity, adenosine triphosphate

## Abstract

Mitochondria are ubiquitous organelles that play a pivotal role in the supply of energy through the production of adenosine triphosphate in all eukaryotic cells. The importance of mitochondria in cells is demonstrated in the poor survival outcomes observed in patients with defects in mitochondrial gene or RNA expression. Studies have identified that mitochondria are influenced by the cell's cytoskeletal environment. This is evident in pathological conditions such as cardiomyopathy where the cytoskeleton is in disarray and leads to alterations in mitochondrial oxygen consumption and electron transport. In cancer, reorganization of the actin cytoskeleton is critical for trans-differentiation of epithelial-like cells into motile mesenchymal-like cells that promotes cancer progression. The cytoskeleton is critical to the shape and elongation of neurons, facilitating communication during development and nerve signalling. Although it is recognized that cytoskeletal proteins physically tether mitochondria, it is not well understood how cytoskeletal proteins alter mitochondrial function. Since end-stage disease frequently involves poor energy production, understanding the role of the cytoskeleton in the progression of chronic pathology may enable the development of therapeutics to improve energy production and consumption and slow disease progression.

This article is part of the theme issue ‘The cardiomyocyte: new revelations on the interplay between architecture and function in growth, health, and disease’.

## 1. Role of mitochondria in energy production

Mitochondria are ubiquitous organelles that are present in most eukaryotic cells [1] where they play a vital role in the production of energy or adenosine triphosphate (ATP) [2]. Mitochondria are also key regulators in cell apoptosis, reactive oxygen species (ROS) production, calcium (Ca^2+^) homeostasis, and contribute to the biosynthesis of amino acids, steroid hormones, haem, lipids and iron–sulfur clusters [2]. The voltage-dependent anion channel (VDAC), a major protein located on the mitochondrial outer membrane (MOM), plays an important role in regulating mitochondrial metabolism, apoptosis and Ca^2+^ signalling [3,4]. VDAC also forms large complexes with other enzymes involved in metabolism, such as cytosolic hexokinase and mitochondrial creatine kinase (mtCK) to facilitate the efficient cycling of metabolites. For example, VDAC provides hexokinase access to mitochondrial ATP for catalysing the phosphorylation of glucose to glucose-6-phosphate [5]. VDAC complex with mtCK at the intermembrane space and adenine nucleotide translocator at mitochondrial inner membrane facilitate high-energy phosphate transfer [6]. Efficient transportation of metabolites such as cytosolic adenosine diphosphate (ADP) and inorganic phosphate, across both mitochondrial membranes, is vital for ATP production within the matrix [7,8]. While the majority (approx. 75%) of ATP is generated via oxidative phosphorylation (OXPHOS) in mitochondria, some is produced via aerobic glycolysis in the cytosol [9]. The energetic yield of ATP per molecule of glucose is substantially lower for aerobic glycolysis (4 moles ATP per mole glucose) compared to OXPHOS (36 moles ATP per mole glucose), therefore, OXPHOS is the more energetically efficient metabolic pathway [10]. Failure of mitochondria to maintain ATP production results in energy deficits and impairments in cell function.

## 2. Role of the cytoskeletal network in cell function

In eukaryotic cells, the cytoskeleton is comprised of microtubules, actin filaments (F-actin) and intermediate filaments. These components differ in stiffness, polarity, modulators, dynamics of their formation and their overall role within cells [11].

Microtubules are the stiffest and are composed of α- and β-tubulin heterodimers [11]. They play critical roles in maintaining cell shape, trafficking of proteins and organelles, and chromosomal segregation during cell division [11]. Microtubules can readily shift between states of rapid elongation by the polymerization of tubulin dimers, as well as rapid depolymerization, thus allowing the dynamic cytoskeleton to reorganize cellular spaces and organelles quickly, for example during the different phases of the cell cycle [11]. F-actins are less stiff in comparison to microtubules and are comprised of two intertwined strands of monomeric globular (G) actin [12]. F-actin also polymerizes and depolymerizes in response to local signalling cues [11], and can provide rigidity and shape to cells, dynamically alter cell shape and initiate cell motility [13]. F-actin uses ATP and generates pulling forces through sliding interactions of actin and myosin filaments [14]. While this mechanism is most notably evident in muscle cells during muscle contraction, it is also applicable to non-muscle cells for other processes such as migration, cytokinesis, modification of cell shape, organization of the extracellular matrix (ECM) and formation of cell–cell/cell–matrix junctions [13]. Intermediate filaments are involved in maintaining the structure and mechanical integrity of cells, particularly bearing tension and creating a supportive scaffold for the internal cellular environment [15]. While intermediate filaments have recently been found to play a role in signal transduction and mechanotransduction, they have less involvement in cell motility compared to microtubules and F-actin [15].

## 3. Cytoskeletal protein regulation of mitochondrial function

Cytoskeletal components work collaboratively to regulate mitochondrial processes of fission/fusion, mitophagy and morphology in response to extracellular stressors [16]. The coordinated action of cytoskeletal components, particularly microtubules and F-actin, are also critical to mitochondrial motility via the distribution and anchoring of the organelles to their appropriate sites in the cell [17]. Specifically, it has been suggested that alterations to the cytoskeleton can affect mitochondria, resulting in functional modifications in the organelle.

While a majority of the previous research linking mitochondria and the cytoskeleton has mostly been investigated in yeast, there have been an increasing number of studies exploring the role of cytoskeletal components in mitochondrial motility and fission/fusion dynamics in mammalian cells [18–21]. These studies have examined the effects of cytoskeletal inhibitors on mitochondrial motility, mitochondrial membrane potential (*Ψ*_m_), morphology and respiration as these can all be indicative of function. We further examine the regulatory role of the cytoskeleton on mitochondrial energetics in different cell types and its implication in disease pathologies.

### (a) Cytoskeletal regulation of mitochondria in cardiomyocytes

In the neonatal heart, energy metabolism occurs predominantly via aerobic glycolysis where the massive proliferation of cardiac myocytes is required for the developing heart [22,23]. This differs from the adult heart in which energy is obtained primarily through OXPHOS, reflecting postnatal energy requirements and increased cardiac efficiency [22,23]. Owing to the high-energy demands of the adult heart, mitochondria play an important role in ATP generation in cardiac myocytes, as well as being involved in Ca^2+^ handling, ROS generation and apoptosis [24]. While cardiac function predominantly relies on ATP generation via OXPHOS, creatine kinase (CK) serves as the heart's primary energy reserve via the phosphocreatine/creatine kinase (PCr/CK) system [25]. This system is critical as it allows rapid generation of high levels of ATP during increased metabolic demand, when ATP usage exceeds its production by OXPHOS, for example during high-intensity physical exercise or ischaemia. In cases of heart failure owing to cardiomyopathy, impairments in energy metabolism have been attributed to reduced myocardial PCr/ATP ratios, indicative of an increased cost of contraction and inefficient metabolism [26].

**Figure 1.**
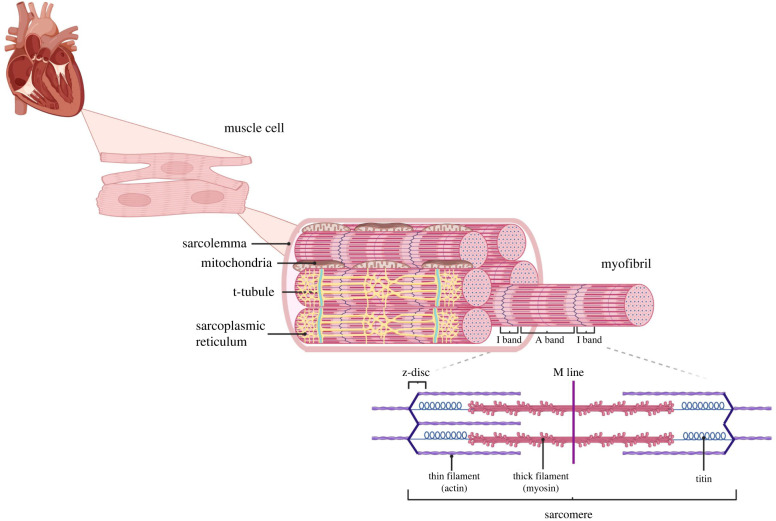
Structure of cardiac myofibrils and the sarcomere. Cardiac myocytes are surrounded by the sarcolemma and contain myofibril bundles that are comprised of repeating contractile units (sarcomeres). Sarcomeres are composed of regions where thin filaments (actin) overlap with thick filaments (myosin) (A-band) and regions of actin only (I-band). Z-discs delineate adjacent sarcomeres and are pulled together during muscle contraction. T-tubules, an extension of the sarcolemma, penetrate through myofibrils and contain various ion channels including the L-type calcium channel (*I*_CaL_). The SR is a specialized structure found within myocytes dedicated to the storage of Ca^2+^ ions. During muscle contraction, Ca^2+^ entry through the *I*_CaL_ triggers Ca^2+^-induced Ca^2+^-release from receptors on the SR. Mitochondria are strategically localized between myofibrils in order to meet the energy requirements of muscle contraction by providing ATP via oxidative phosphorylation (OXPHOS). Adapted from ‘Myofibril Structure’, by BioRender.com (2022). Retrieved from https://app.biorender.com/biorender-templates.

The cytoskeletal network forms the scaffold of cardiac muscle cells and extends from the plasma membrane to z-discs of the sarcomere, as well as traversing organelles including t-tubules, sarcoplasmic reticulum (SR) and mitochondria (figure 1). The positioning of sarcomeres within adult cardiomyocytes is tightly regulated by the cytoskeletal network and the SR.

Cardiac L-type Ca^2+^ channel (*I*_CaL_) activation and inactivation kinetics are vital to the processes of excitation and contraction. The *I*_CaL_ is physically anchored to F-actin via the channel's auxiliary β2-subunit and F-actin-associated protein AHNAK [27]. Mitochondria are also structurally associated with F-actin via mitochondrial docking proteins [28]. *I*_CaL_ activation kinetics can modulate mitochondrial function through this physical association, as observed in isolated cardiac myocytes, where *I*_CaL_ activation with *I*_CaL_ agonist BayK(-) directly results in increased *Ψ*_m_ under Ca^2+^-free experimental conditions [29]. This increased response is attenuated in the presence of the F-actin depolymerizing agent latrunculin A [29]. As the response is dependent on an intact cytoskeleton, this indicates that cytoskeletal changes can modulate mitochondrial energetics through alterations in *I*_CaL_ kinetics [29,30]. This is consistent with other studies in which the dissociation of microtubules or depolymerization of F-actin alters *I*_CaL_ inactivation rate [30].

A pivotal study undertaken by Saks *et al.* [31] demonstrated the existence of a diffusion barrier for ADP in MOM. Specifically, they found that respiration of isolated mitochondria is characterized by an apparent *K*_m_ for exogenous ADP of approximately 10-fold lower than in permeabilized cells and that mild trypsin treatment of cells results in an increase of MOM permeability up to the level of isolated mitochondria [32]. These surprising observations led them to suggest the existence of the so-called ‘Factor X’—an intracellular cytoplasmic component controlling the permeability of MOM for ADP in cardiac muscle cells *in vivo*. They proposed that this factor was linked to the cytoskeleton as it was still present in tissue homogenate where it seemed to be connected to mitochondria and other structures but was absent after centrifugation in isolated mitochondria [31]. More than 10 years later this factor was identified as free dimeric tubulin—a building block of microtubules—and was confirmed to regulate ATP fluxes through VDAC *in vitro* [33,34]. The importance of this interaction is that VDAC transiently blocked by tubulin is impermeable for ATP or ADP. This phenomenon arises owing to the highly negatively charged tubulin C-terminal tail (either α- or β-tubulin) [35] entering the net positive pore of VDAC, which reverses the net charge of the pore interior, creating electrostatic and steric barriers for ATP and other negatively charged mitochondrial metabolites. Experiments using isolated cardiac mitochondria demonstrated that dimeric tubulin restricts the availability of ADP for OXPHOS and reduces mitochondrial respiration [33,36], further confirming that cytoskeletal proteins can regulate mitochondrial respiration by interacting directly with VDAC. According to their model, VDAC-cytoskeleton interaction selectively restricts channel permeability for ATP and ADP, but not for creatine or phosphocreatine, thus shifting the energy transfer to the PCr/CK pathway. Interestingly, in cancerous non-beating HL-1 cells of cardiac phenotype, where both β2 tubulin isoform and mtCK are absent, the apparent *K*_m_ for exogenous ADP is low [37]. Some cancer cells show increased β3 tubulin expression [38] which may play a role in regulating VDAC activity, but more studies are needed to confirm its over-expression in HL-1. A hypothetical model has been proposed which suggests that hexokinase-II replaces β2 tubulin in binding to VDAC and switch the energy transfer to the Warburg–Pedersen pathway [39–41]. However, more studies are needed to confirm the Warburg–Pederson pathway.

**Figure 2.**
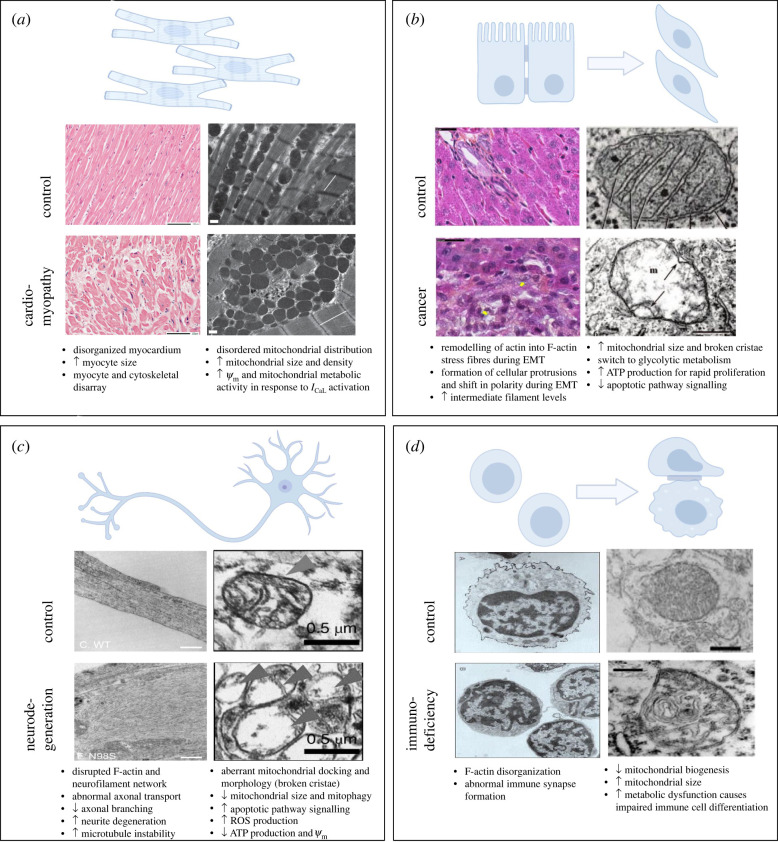
Mitochondria and the cytoskeletal network in health and disease. Cytoskeletal and mitochondrial abnormalities are predominant features in many disease states including neurodegeneration, cardiomyopathy, cancer and immunodeficiencies. (*a*) In cardiomyopathies, myocyte disorganization and cytoskeletal disarray are key features, along with disordered mitochondrial distribution, increased metabolic activity and *Ψ*_m_ (scale bars in haematoxylin and eosin stained sections (left) and transmission electron microscopic images (right) represent 100 µM and 0.5 µM, respectively) [29,43]. (*b*) Epithelial to mesenchymal transition (EMT) in cancer cells is driven by cytoskeletal remodelling with tumour growth and metastasis being associated with aberrant mitochondrial morphology, increased ATP production through metabolic reprogramming to glycolysis and decreased apoptotic signalling (scale bars in haematoxylin–phloxine–saffron stained sections (left), and uranyl acetate/lead citrate stained sections (right) represent 25 µM, and 0.33 µM, respectively) [44,45]. (*c*) In neurodegeneration, disruption to F-actin, microtubules and neurofilaments are associated with impaired axonal transport, decreased branching and increased neurite degeneration (scale bars in electron micrographs represent 2 µM (left) and 0.5 µM (right), respectively) [46]. Aberrant mitochondrial transport, docking and morphology is also linked to increased apoptotic signalling and ROS production as well as decreased ATP production and *Ψ*_m_ in neurons [47]. (*d*) F-actin disorganization in lymphocytes is associated with impaired immune synapse (IS) formation in immunodeficiencies, in which impaired mitochondrial biogenesis and metabolic dysfunction are also prominent (transmission electron microscopic images obtained at ×18 400 (left) ×25 000 (right) magnification—scale bar units were not reported [48,49].

Many cardiomyopathies exhibit alterations in cytoskeletal proteins, as well as disturbances in mitochondrial oxygen consumption and electron transport. In hypertrophic cardiomyopathy (HCM), a highly disorganized cytoskeleton arises owing to mutations in sarcomeric proteins [42] (figure 2*a*). This cytoskeletal disarray is linked to alterations in energy metabolism, which occur prior to the onset of pathological features, such as altered cardiac contractility, left-ventricular hypertrophy and interstitial fibrosis [42]. In mitochondria that have been isolated from HCM hearts, normal respiration and complex activity are observed although mitochondrial gene expression, morphology and number are altered [42], emphasizing the role of cytoskeletal proteins (and the intracellular environment) in regulating mitochondrial function. In murine models of Duchenne muscular dystrophy (DMD), the absence of cytoskeletal protein dystrophin in the heart leads to alterations in *Ψ*_m_, mitochondrial electron transport and contractile dysfunction [29]. Owing to the physical association of the *I*_CaL_ and mitochondria through the cytoskeletal network, we can see how in the case of DMD, the disrupted cytoskeletal architecture owing to the absence of dystrophin, leads to depressed mitochondrial metabolic activity, indicating a regulatory role of the cytoskeleton in mitochondrial function. Studies have also suggested the role of the mitochondrial channel VDAC in these processes, as the interaction of the hexokinase N-terminal peptide with VDAC mimics the effects of *I*_CaL_ agonist BayK(-) on *Ψ*_m_ [29,42]. Mitochondrial abnormalities are also observed in many desmin-related cardiomyopathies. Knock-out murine models exhibit impairments in mitochondrial structure as early pathological features, occurring prior to any other cardiac dysfunction [50]. Mitochondrial structural abnormalities such as damaged cristae lead to oxidative stress, altered metabolic activity, cardiac myocyte death and heart failure [51] (figure 2*a*). Desmin is primarily found at the mitochondria-associated membrane between the SR and mitochondria, where it associates with VDAC [51], further suggesting a role for the cytoskeleton in regulating Ca^2+^ and metabolite trafficking through VDAC in mitochondria.

### (b) Cytoskeletal regulation of mitochondria in cancer cells

The Warburg effect, initially described in the 1920s, suggests that cancer cells have a modified cellular metabolism, in which they favour aerobic glycolysis to mitochondrial OXHPOS for energy production [10,52]. Mammalian cell growth is controlled by systems that prevent aberrant proliferation and only uptake and metabolize nutrients from their environment when stimulated by specific growth factors [53]. While aerobic glycolysis is inefficient for ATP generation, it is proposed that cancer cells and other proliferating cells use this mode of metabolism to promote nutrient uptake, and fuel cell growth [53]. Although it was previously believed that this shift in metabolism was owing to defective mitochondria and subsequent impaired respiration, it is now known that mitochondrial function is not impaired in most cancer cells, as demonstrated by mitochondria isolated from tumours that maintain effective *Ψ*_m_ formation and activity of electron transport chain components [53,54]. By contrast to therapeutic targeting of mitochondria in neurodegenerative and cardiac diseases where the primary goal is to prevent cell death, therapeutic targeting of mitochondria in cancer has the specific goal of inducing apoptosis to cause death to malignant cells.

Epithelial to mesenchymal transition (EMT), a crucial cellular process for embryonic development and wound tissue healing, is also implicated in the progression of diseases, such as cancer metastasis [55]. F-actin, microtubules and intermediate filaments all play essential roles in activating EMT and promoting cancer metastasis [56]. Epithelial cells have apical membranes that contact the environment and basal membranes that are anchored to the ECM [56]. Dynamic remodelling of actin into F-actin stress fibres during EMT is associated with alterations in cell polarity from apical–basal to front-rear polarity, which triggers a transition in these cells [56] (figure 2*b*). The shift in polarity involves morphological changes and restructuring of the cell's attachment to the ECM, to form spindle-like mesenchymal phenotypes with various leading-edge protrusions that drive migration and invasion [56].

In cancer, growth factors including transforming growth factor *β* and epithelial growth factor induce signalling pathways such as Wnt and Notch that induce transcription programme switching in EMT [57]. EMT can also be induced by the under-expression of proteins associated with cell–cell adhesion and ECM, as well as tumour microenvironment factors such as oxidative stress, alterations in metabolic activity and ECM stiffness [58]. Many studies suggest that ECM stiffness is important in cancer cells as they have mechanosensing properties and mechanotransduction feedback loops that are implicated in cytoskeletal rearrangement and cell differentiation processes [58]. Integrins are transmembrane proteins that link the ECM to the cell's cytoskeleton. The effects of integrin-mediated cues on mitochondrial migration and metabolism have been well established in cancer cells [59]. Furthermore, detachment of the ECM by integrins is associated with the disruption of focal adhesions and cytoskeleton, leading to downstream activation of pro-apoptotic signalling pathways [60].

Free dimeric tubulin is also increased in proliferative cancer cells as it is required for spindle formation during cell division [61]. Studies have demonstrated that dimeric tubulin can alter mitochondrial function through VDAC by transiently blocking VDAC, as shown in *in vitro* experiments as well as knock-down VDAC cell models [33,34], and subsequently inhibits transport of respiratory substrates through the channel, leading to the maintenance of lower ATP/ADP ratios, stimulation of glycolytic metabolism and decreased *Ψ*_m_ [54,62]. As VDAC is the only means of metabolite flux through the MOM, it is conceivable that its conductance mediates mitochondrial metabolism [54]. VDAC-tubulin interaction is suggested to play a role in the suppression of mitochondrial metabolism, thereby triggering the switch to glycolytic metabolism that is associated with the Warburg effect and cell proliferation in cancer cells [54] (figure 2*b*). Over-expression of tubulin isoforms appears to facilitate cancer progression and chemo-resistance [63] through enabling the glycolytic switch, cancer cell proliferation and downregulation of apoptotic pathways, thereby providing further support for the regulation of mitochondrial energetics by cytoskeletal proteins.

### (c) Cytoskeletal regulation of neuronal mitochondria

Although the brain only accounts for 2% of body mass, it has an incredibly high metabolic demand and uses approximately 20–25% oxygen consumption at rest [64]. Mitochondria provide the energy to meet demanding processes including maintaining resting membrane potentials, regulating axonal and dendritic development, peripheral axonal regeneration, synaptic function and nerve signalling [64]. As with other cell types, neurons rely on mitochondria for Ca^2+^ homeostasis, steroid synthesis, apoptosis and ROS generation [65]. Many neurodegenerative diseases that are characterized by gradual neuronal loss and synaptic dysfunction are also associated with abnormal energy metabolism and mitochondrial impairments [65,66].

During embryonic development, neurons are dependent on the cytoskeleton for processes such as cell proliferation, differentiation, migration, axonal guidance and dendrite arborization [67]. These processes are heavily reliant on the microtubule network for establishing cell polarity and aiding neural migration to ensure appropriate connections and synapses are established and maintained throughout development [67].

The cytoskeletal network is just as important during adulthood, with evidence indicating cytoskeletal disruption to be a prominent feature of many neurodegenerative diseases [68] (figure 2*c*). In particular, alterations in microtubule stability and dysregulation of microtubule- or actin-associated proteins (MAPs or AAPs) appear to be key players, leading to downstream impacts on microtubule and F-actin dynamics, axonal transport, neurite outgrowth, intracellular trafficking and synaptic plasticity [68]. For example, in Alzheimer's disease (AD), hyperphosphorylated tau leads to microtubule disorganization and generation of bundles of abnormal filaments (neurofibrillary tangles) that ultimately result in dystrophic neurites, synaptic loss and neuronal cell death [69]. Huntington's disease and Parkinson's disease (PD) are also associated with abnormal processing of tau, leading to microtubule instability, which is associated with both axonal impairment and neurite degeneration *in vitro* [70].

Mitochondrial localization and distribution throughout neurons are heavily reliant on an intact cytoskeleton as ATP has a slow diffusion rate [71]. Therefore, efficient mitochondrial trafficking and docking are crucial for ATP production at energy-demanding sites [65]. It is now evident that abnormalities in mitochondrial localization lead to impairments in their dynamics and functioning [72].

In neurons, specifically within the axonal growth cone and at pre-synaptic terminals, microtubules, F-actin and neurofilaments are involved in moving mitochondria along and anchoring them throughout the axon and at synapses [73]. Microtubules serve as tracks along which mitochondria move in an anterograde or retrograde manner towards the pre-synaptic terminal or the nucleus, respectively [74]. Neuronal axonal transport also involves interactions between ATP-dependent motor proteins (such as kinesins and dyneins) and motor adaptor proteins (such as TRAK 1/2, milton, KIF5, syntabulin and dynactin) [74]. Motor adaptor proteins interact closely with the cytoskeleton and disruptions to these proteins result in alterations to mitochondrial transport in dendrites and axons, as well as subsequent disruption to synaptic transmission and mitochondrial dynamics [75,76], implicated in neurological conditions such as AD, amyotrophic lateral sclerosis, PD and Charcot–Marie–Tooth disease [73].

F-actin also plays a crucial role in the docking of neuronal mitochondria along axons and at pre-synaptic terminals where it is densely localized [77]. *In vitro* studies where neuronal F-actin is disrupted, demonstrate increased velocity of motile mitochondria leading to reduced or aberrant docking [78]. Loss of actin-related proteins (ARPs), a class of proteins similar to the conventional actin, also lead to improper mitochondrial accumulation in axon terminals [74]. As transport and localization of other cargo such as lysosomes and peroxisomes remain unaffected in these instances, it emphasizes an important role of F-actin and ARPs in regulating proper mitochondrial anchoring at specific subcellular sites [74].

Disruption to cytoskeletal components and subsequent impairments to fission/fusion dynamics alters mitochondrial morphology and downstream functions such as neurotransmitter release, vesicle recycling and synaptic plasticity [65] (figure 2*c*). *In vitro* models of AD over-expressing MAP tau, results in a shift towards excessive fission, reduction of dynamin-related protein 1 (DRP1) recruitment, mitochondrial fragmentation and clustering abnormalities [79]. Animal models of AD exhibit neuronal mitochondria with highly heterologous morphology, in particular fragmented or elongated mitochondria, as well as a significant reduction in fission-associated proteins such as OPA1 [76]. Fragmented mitochondria are similarly observed in models of PD [80]. AD patients also demonstrate a high number of mitochondria with broken cristae compared to age-matched controls, [81], mirroring *in vitro* studies where fragmented mitochondria are also observed in neurons over-expressing APP [82]. Mitochondrial fragmentation and disruption of mitochondrial morphology have been linked to impaired mitochondrial motility and bioenergetics in the literature, and can trigger apoptotic pathways, thereby facilitating neurodegeneration [83]. Furthermore, abnormalities in mitochondrial fusion can lead to swelling and aggregation of mitochondria resulting in larger diameters that prevent their entry into smaller distal neurites, impacting mitochondrial localization in energy-demanding sites and giving rise to neuronal deficits [84].

### (d) Cytoskeletal regulation of mitochondria in lymphocytes

The innate immune response is a general system that mediates rapid inflammatory responses through non-specific physical/chemical defences, whereas the adaptive immune response is a specialized system that emerges over time after initial infection, through antigen-specific responses [85]. Lymphocytes make up a large subpopulation of immune cells including T cells, B cells and natural killer cells, with the adaptive immune response being directed largely by T and B cells [85].

Naive immune cells are metabolically quiescent, with minimal nutrient uptake, and as such primarily use the efficient OXPHOS system to generate ATP [86]. Antigen-activated T cells, however, undergo rapid proliferation, requiring a considerable amount of energy and cellular resources [86]. Similar to cancer cells, activated T cells exhibit the Warburg effect, shifting from the tricarboxylic acid cycle and OXPHOS to aerobic glycolysis for ATP generation, a process that is stimulated by growth factor cytokines [86]. Metabolic dysfunction can impact immune cell fate, leading to a lack of specific immune cells or impairments in immune cell differentiation, which is implicated in immune deficiency disorders [87].

The cytoskeleton plays an important role in normal immunological function, with dynamic rearrangement of the cytoskeleton an essential process for appropriate adhesion, migration, activation and proliferation of lymphocytes [88]. External environmental cues can activate signalling pathways that trigger T-cell recruitment to sites of damage and provoke changes in T-cell morphology and motility via cytoskeletal rearrangement [88]. These cues include interactions between chemokines with chemokine receptors on T cells, adhesion molecules and their ligands on other lymphocytes, as well as mechanical forces via the ECM [88]. Alterations in the ability of cytoskeletal components to undergo remodelling during differentiation and activation have resulted in immune deficiency, auto-immunity and auto-inflammatory disease [88].

In lymphocytes, fission/fusion dynamics and motility are vital in maintaining a healthy mitochondrial network. The cytoskeleton and the endoplasmic reticulum interact with the mitochondrial network to regulate these processes. GTPases are also involved in morphological changes and fission/fusion processes, including mitofusins, OPA1 and DRP1 [89]. Impairments in mitochondrial fission and fusion leads to poor mixing of mitochondrial DNA (mtDNA) and an accumulation of high levels of pathogenic mtDNA which can impact respiratory functioning in T cells [89]. Furthermore, improper fission/fusion dynamics can also affect mitochondrial morphology and subsequent localization [89] (figure 2*d*). Specifically, smaller mitochondria can move more easily, whereas larger mitochondrial networks are more difficult to move along cytoskeletal structures. It has recently been shown in T cells that DRP1 silencing inhibits mitochondrial motility and localization at the immune synapse (IS) [90]. Localization of mitochondria at the IS is crucial as it facilitates Ca^2+^ influx and ATP production to be appropriately maintained across the plasma membrane needed for downstream activations such as T-cell protrusion, polarization and migration [91]. IS assemblage is closely partnered with cytoskeletal-dependent mitochondrial redistribution towards the T-cell/antigen-presenting cell interface and appropriate localization of mitochondria is fundamental in regulating metabolic adaptations in processes of T-cell differentiation, migration and activation [92].

## 4. Conclusion

Mitochondrial dysfunction and poor energy production are characteristic features of many end-stage diseases. Similarly, cytoskeletal disruption and disorganization are characteristic features of many pathological conditions. It is now well-recognized that cytoskeletal proteins are important for the localization and physical tethering of mitochondria to areas of metabolic demand including the A band of sarcomeres in cardiac myocytes, the growth cone of neuronal axons, the leading edge of migrating cancer cells and uropods in migrating lymphocytes. Therefore, it is critical to expand our understanding of the role of the cytoskeleton in the progression of chronic pathology and how cytoskeletal proteins alter mitochondrial function. Here, we focus on cytoskeletal abnormalities and how they affect mitochondrial morphology, fission/fusion dynamics, motility and distribution in a variety of cell types. Cytoskeletal regulation of mitochondrial morphology, organization and distribution in cells is inextricably linked to their main function of energy production and transfer—the OXPHOS. The cytoskeletal abnormalities can subsequently lead to impairments in mitochondrial respiration and activation of apoptotic pathways, thereby accelerating disease progression. To this end, the multiple complexes of mitochondria with cytoskeleton proteins may represent a potential therapeutic target in the management of various human diseases.

## Data Availability

This article has no additional data.
